# Postharvest Calcium Chloride Treatment Strengthens Cell Wall Structure to Maintain Litchi Fruit Quality

**DOI:** 10.3390/foods12132478

**Published:** 2023-06-25

**Authors:** Xiaomeng Guo, Qiao Li, Tao Luo, Dongmei Han, Difa Zhu, Zhenxian Wu

**Affiliations:** 1 College of Horticulture, South China Agricultural University, Guangzhou 510642, China; guoxm_scau@163.com (X.G.); liqiao@stu.scau.edu.cn (Q.L.); luotao0502@scau.edu.cn (T.L.); dfzhu@stu.scau.edu.cn (D.Z.); 2 Guangdong Provincial Key Laboratory of Postharvest Science of Fruits and Vegetables, Engineering Research Center of Southern Horticultural Products Preservation, Ministry of Education, Guangzhou 510642, China; 3 Key Laboratory of Biology and Genetic Improvement of Horticultural Crops (South China), Ministry of Agriculture and Rural Affairs, College of Horticulture, South China Agricultural University, Guangzhou 510642, China; 4 Institute of Fruit Tree Research, Guangdong Academy of Agricultural Sciences, Key Laboratory of South Subtropical Fruit Biology and Genetic Resource Utilization, Ministry of Agriculture, Guangzhou 510640, China; handongmei@gdaas.cn

**Keywords:** litchi, postharvest, calcium chloride, cell wall, pericarp browning, pulp, decay

## Abstract

Litchi (*Litchi chinensis* Sonn.) fruit deterioration occurs rapidly after harvest and is characterized by pericarp browning, pulp softening, and decay. In this study, we found that calcium chloride (CaCl_2_) treatment (5 g L^−1^ CaCl_2_ solution vacuum infiltration for 5 min) affected the cell wall component contents and cell wall-degrading enzyme activities of litchi fruit during storage at room temperature. CaCl_2_ treatment significantly increased the contents of Ca^2+^ and cellulose, while it decreased the water-soluble pectin content, and the activities of polygalacturonase, *β*-galactosidase, and cellulase in the litchi pericarp. Meanwhile, the treatment resulted in significantly increased contents of Ca^2+^, water-soluble pectin, ionic-soluble pectin, covalent-soluble pectin and hemicellulose, and upregulated activities of pectinesterase and *β*-galactosidase, while significantly decreasing the activities of polygalacturonase and cellulase in litchi pulp. The above results indicate that CaCl_2_ treatment strengthened the cell wall structure of litchi fruit. More importantly, the enzymatic browning of the pericarp, softening of the pulp, and disease incidence were delayed. The treatment had a more pronounced effect on the pericarp than on the pulp. We consider CaCl_2_ treatment to be a safe and effective treatment for maintaining the postharvest quality of litchi fruit.

## 1. Introduction

Litchi (*Litchi chinensis* Sonn.) is a fruit indigenous to China [1]. China is the leading litchi producer in the world [2]. Pericarp browning, pulp deterioration, and decay are major postharvest problems that significantly reduce the commodity value of litchi fruit, and limit the development of the industry [3]. In recent years, safe and effective treatments have been developed to meet the increasing demand for maintaining the postharvest quality of litchi fruit. Recently, many substances, such as proanthocyanidin [4], melatonin [5], apple polyphenols [6], fluopyram [7], methyl jasmonate [8], benzothiadiazole [9], and α-lipoic acid [10] have been used to treat postharvest litchi fruit and achieved good effects.

Calcium treatment is a safe treatment that effectively delays fruit ripening and senescence, and reduces the incidence of physiological and pathological diseases. Specifically, Ca^2+^ could maintain or strengthen the structure and function of the cell wall and membrane, reduce the respiration and ethylene synthesis of fruit, and regulate enzyme activity and the senescence-related metabolism [11]. Calcium treatment, especially calcium chloride (CaCl_2_) treatment, has been shown to have a positive effect on the postharvest preservation of many fruits. For example, CaCl_2_ treatment effectively maintains qualities as the firmness, color, sugar and acid contents of tomato [12], date [13], and apple [14], and enhances the antioxidation of grape [15], jujube [16,17], pear [18], banana [19], blackberry [20], and papaya [21]. CaCl_2_ treatment also significantly maintains or strengthens the cell wall structure of grape [22], apple [23], red raspberry [24], papaya [25], melon [26], apricot [27], and sweet cherry [28], and improves the cold resistance of apple [29], peach [30], jujube [16], loquat [31], and banana [32], in addition to reducing the decay of melon [26,33]. However, the application of CaCl_2_ treatment in the postharvest preservation of litchi fruit has not been reported.

On the one hand, the litchi pericarp is very thin, and susceptible to browning and decay due to mechanical damage, disease infection, and water loss. On the other hand, litchi pulp softens quickly after the harvest due to the degradation of cell wall polysaccharides, especially when stored at room temperature [34]. Considering the widely reported effectiveness of CaCl_2_ treatment in improving postharvest resistance and maintaining fruit quality, we tested the effect of this treatment on the postharvest storage of litchi fruit and analyzed the related physiological and biochemical indices. We believe that CaCl_2_ treatment could be a promising method to solve the above-mentioned postharvest problems of litchi fruit. This study aims to provide a CaCl_2_ treatment method for maintaining the quality of litchi fruit stored at room temperature and to investigate the mechanism of CaCl_2_ treatment on litchi fruit.

## 2. Materials and Methods

### 2.1. Plant Material and Treatments

Commercially mature (bright red pericarp) litchi fruits (cultivar ‘Guiwei’) were obtained from a commercial orchard in Guangzhou, China. Approximately 600 healthy litchi fruits were divided into two groups. Each of the two groups of fruits was vacuum-infiltrated (−33 kPa) in 20 L of water (control), or 5 g L^−1^ CaCl_2_ (Damao, China) solution (CaCl_2_ treatment) for 5 min at 20 °C, and then air-dried. The fruits were stored at room temperature (25 °C ± 2 °C) in polyethylene terephthalate trays (approximately 20 fruits per tray), and packaged with 0.01 mm polyvinyl chloride film. A total of 60 litchi fruits per group were analyzed in triplicate on days 0, 1, 3 and 5. Meanwhile, the pericarp and pulp of 20 litchi fruits were divided into three replicates, frozen in liquid nitrogen, and stored at −80 °C.

### 2.2. Determination of Pericarp Browning Index and Decay

In total, 60 fruits (at the last point in time, there was a total of 100 fruits) were randomly selected for each treatment to evaluate the pericarp browning index and decay according to the method described by Guo et al. [3]. The details are shown in the Supplementary Materials.

### 2.3. Determination of Calcium Content

The calcium content was determined according to the method described by Erdogan et al. with modifications [35]. A total of 0.2 g of oven-dried pericarp or 0.4 g of oven-dried pulp samples and 5 mL of concentrated HNO_3_ were added to the digestion vessel overnight. Then, 3 mL of 30% H_2_O_2_ was added for microwave digestion. After cooling the digestion vessel to room temperature, the contents were transferred to a volumetric flask, diluted to 50 mL or 25 mL with deionized water, and filtered with filter paper for determination using a flame atomic absorption spectrometer (Spectr AA 220FS, Varian, Palo Alto, CA, USA).

### 2.4. Determination of Cell Wall Component Contents

The cell wall components, including water-soluble pectin (WSP), ionic-soluble pectin (ISP), covalent-soluble pectin (CSP), hemicellulose, and cellulose, were extracted and determined based on the method described by Lin et al. [36]. Briefly, 5 g of ground samples of pericarp or pulp were boiled with 80% ethanol, and centrifuged repeatedly to inactivate enzymes and remove sugars. The residues were washed three times with chloroform/methanol (1:1), then dissolved in 90% dimethyl sulfoxide overnight, and centrifuged until all starch was removed. The residues were thoroughly dried at 40 °C as cell wall components after washing three times with acetone.

Cell wall components were immersed in ultrapure water for 6 h to obtain WSP. The water-insoluble residues were immersed in 50mM sodium acetate buffer (pH 6.8) containing 1M 1,2-cyclohexanediaminetetraacetic acid (CDTA) for 6 h to obtain ISP. The CDTA-insoluble residues were immersed in 50 mM sodium carbonate (Na_2_CO_3_) containing 20 mM sodium borohydride (NaBH_4_) for 6 h to obtain CSP. The Na_2_CO_3_-insoluble residues were immersed in 50 mM sodium hydroxide containing 100 mM NaBH_4_ for 6 h to obtain hemicellulose. The residues were washed with 0.1 M potassium hydroxide (KOH) and 8 mM sodium sulfite three times, and then immersed in 8 M KOH for 6 h. The KOH-insoluble residues were washed three times with ethanol and thoroughly dried at 40 °C to obtain cellulose.

The contents of WSP, ISP and CSP were determined by the carbazole method with galacturonic acid as standard. The contents of cellulose and hemicellulose were determined by the anthrone method with glucose as standard.

### 2.5. Determination of Cell Wall-Degrading Enzyme Activities

The crude enzyme was extracted according to the method described by Lin et al. with modifications [36]. A total of 3 g samples of pericarp or pulp ground with 15 mL of extraction buffer containing 50 mmol L^−1^ sodium acetate buffer (pH 5.2), 100 mmol L^−1^ NaCl, 5 mmol L^−1^ dithiothreitol, and 5% (*w*/*v*) polyvinylpyrrolidone. The supernatant was used as the crude enzyme.

Pectinesterase (PE) activity was determined according to the method described by Lin et al. with modifications [36]. The reaction mixture contained 1.5 mL of crude enzyme and 5 mL of 1 % (*w*/*v*) pectin. After incubation at 37 °C for 30 min, the reaction was stopped by boiling for 5 min. The pH of the reaction solution was titrated to 7.4 with 0.01 mol L^−1^ NaOH. The amount of enzyme consumed 1 μmol of NaOH per hour was defined as one unit of PE activity. Polygalacturonase (PG) activity was determined according to the method described by Chen et al. with modifications [37]. The reaction mixture contained 1.3 mL of 50 mmol L^−1^ sodium acetate buffer (pH 4.5), 0.5 mL of 10 g L^−1^ polygalacturonic acid, and 0.2 mL of crude enzyme. The mixture was incubated at 37 °C for 1 h, and 1.5 mL of 3,5-dinitro salicylic acid reagent was added and boiled for 5 min to stop the reaction. After cooling on ice, the absorbance was measured at 540 nm. The amount of enzyme produced 1 μg of galacturonic acid per hour was defined as one unit of PG activity. Cellulase (Cx) activity was determined according to the method described by Chen et al. with modifications [37]. The reaction mixture contained 1.8 mL of 10 g L^−1^ carboxymethyl cellulose and 0.2 mL of crude enzyme. Cx activity was determined to be the same as PG activity. The amount of enzyme produced 1 mg of glucose per hour was defined as one unit of cellulose activity. The *β*-galactosidase (*β*-Gal) activity was determined according to the method described by Lin et al. with modifications [36]. The reaction mixture contained 1 mL of crude enzyme and 1 mL of 5 mmol L^−1^ *p*-nitrophenyl-*β*-D-galactopyranoside. After incubating at 37 °C for 30 min, the reaction was stopped by adding 4 mL of 1 mol L^−1^ Na_2_CO_3_. Absorbance was measured at 400 nm. The amount of enzyme produced 1 μmol of *p*-nitrophenol per hour was defined as one unit of *β*-Gal activity. The protein content was determined according to the method established by Bradford [38]. The activity of each enzyme was expressed as U mg^-1^ protein.

## 3. Results

### 3.1. Effects of CaCl_2_ Treatment on Pericarp Browning and Decay of Litchi Fruit

**Figure 1:**
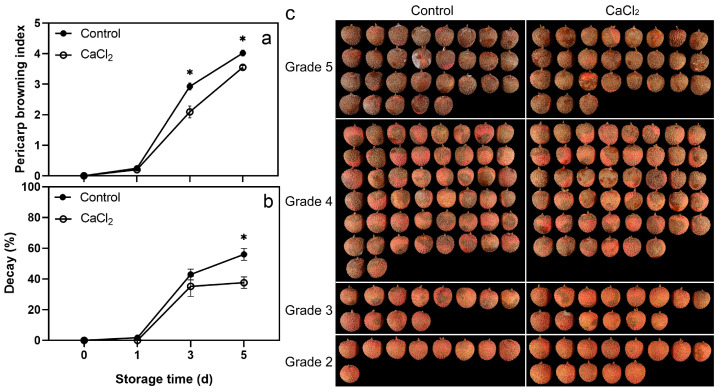
Effects of CaCl_2_ treatment on pericarp browning index (**a**) and decay (**b**) of litchi fruit. Detailed appearance of control and CaCl_2_-treated fruits according to their grades of pericarp browning on day 5 (**c**). Asterisks indicate significant differences between CaCl_2_-treated and control fruits (* *p* < 0.05). The data are presented as the mean ± SEM (standard error of the mean) of three replicates.

The pericarp browning index and decay of litchi fruit showed an increasing trend with storage. The pericarp browning index of CaCl_2_-treated fruit was significantly lower than that of the control on days 3–5 (Figure 1a,c). The decay increased rapidly during days 1–3, and the decay of CaCl_2_-treated fruit was significantly lower than that of the control on day 5 (Figure 1b).

### 3.2. Effects of CaCl_2_-Treated on Cell Wall Component Contents and Cell Wall-Degrading Enzyme Activities in Litchi Pericarp

**Figure 2:**
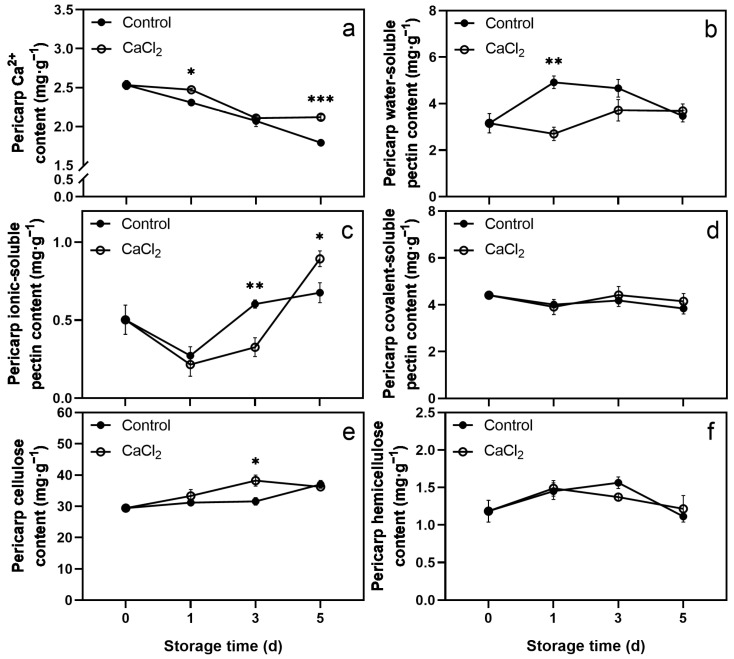
Effects of CaCl_2_ treatment on the contents of Ca^2+^ (**a**), water-soluble pectin (**b**), ionic-soluble pectin (**c**), covalent-soluble pectin (**d**), cellulose (**e**), and hemicellulose (**f**) in litchi pericarp. Asterisks indicate significant differences between CaCl_2_-treated and control fruits (* *p* < 0.05, ** *p* < 0.01, *** *p* < 0.001). The data are presented as the mean ± SEM of three replicates.

The Ca^2+^ content in both CaCl_2_-treated and control pericarp decreased during storage. The Ca^2+^ content in the CaCl_2_-treated pericarp was significantly higher than that in the control on days 1 and 5 (Figure 2a).

The WSP content in the control pericarp increased rapidly on the first day, then showed a slight decrease from day 1 to day 5. In contrast, the WSP content in the CaCl_2_-treated pericarp slightly decreased on the first day of storage and then increased slightly. The WSP content in the CaCl_2_-treated pericarp was significantly lower than that in the control on day 1 (Figure 2b). A quick decline in the ISP content was observed on the first day, whereas later, it increased continuously. A significantly lower ISP content was observed in CaCl_2_-treated pericarp than in the control on day 3. However, a significantly higher ISP content was observed in CaCl_2_-treated pericarp than in the control on day 5 (Figure 2c). The cellulose content increased slightly during storage. Compared to the control, the cellulose content in the CaCl_2_-treated pericarp was significantly higher on day 3 (Figure 2e). CaCl_2_ treatment did not affect the contents of CSP and hemicellulose in the pericarp (Figure 2d,f).

**Figure 3:**
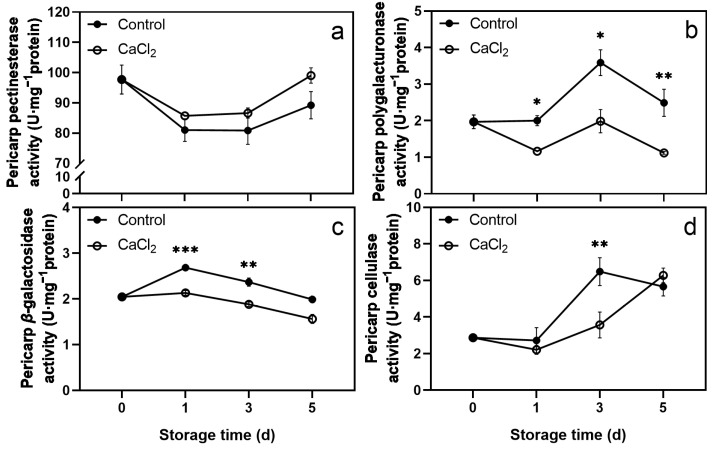
Effects of CaCl_2_ treatment on the activities of pectinesterase (**a**), polygalacturonase (**b**), *β*-galactosidase (**c**), and cellulase (**d**) in litchi pericarp. Asterisks indicate significant differences between CaCl_2_-treated and control fruits (* *p* < 0.05, ** *p* < 0.01, *** *p* < 0.001). The data are presented as the mean ± SEM of three replicates.

The CaCl_2_ treatment significantly inhibited the activities of PG, *β*-Gal, and Cx in the pericarp. Lower activities of PG, *β*-Gal, and Cx were observed in the CaCl_2_-treated pericarp compared to the control pericarp throughout storage (Figure 3b–d). However, CaCl_2_ treatment did not affect the PE activity in the pericarp (Figure 3a).

The PG activity in the control pericarp increased rapidly from day 1 to day 3, and then decreased. The PG activity in the CaCl_2_-treated pericarp was always significantly lower than that in the control (Figure 3b). The *β*-Gal activity in the control pericarp rose on the first day, and then declined, whereas the β-Gal activity in the CaCl_2_-treated pericarp showed a downward trend and was lower than that in the control pericarp over the storage period (Figure 3c). A sharp increase in the Cx activity was observed in the control pericarp from day 1 to day 3. The Cx activity in the CaCl_2_-treated pericarp rose continuously during days 1–5 and was significantly lower than that in the control on day 3 (Figure 3d).

### 3.3. Effects of CaCl_2_-Treated on Cell Wall Component Contents and Cell Wall-Degrading Enzyme Activities in Litchi Pulp

**Figure 4:**
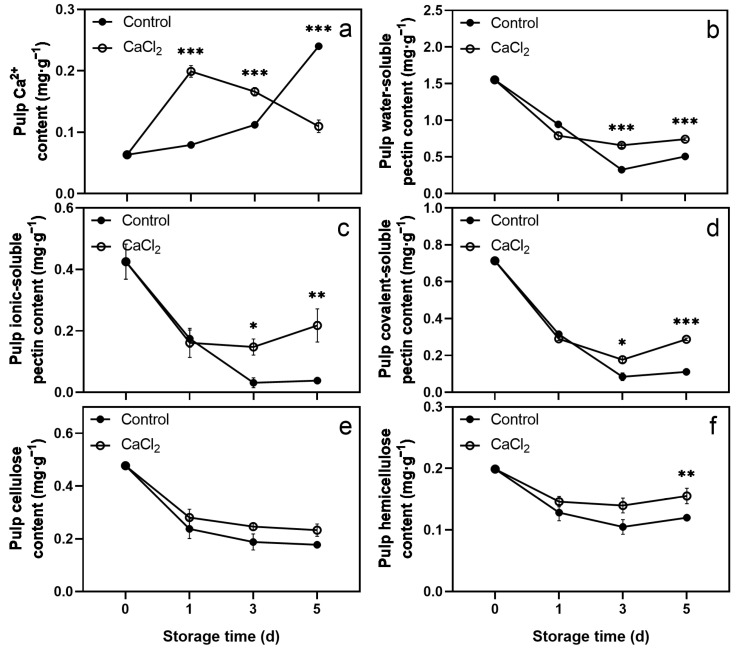
Effects of CaCl_2_ treatment on the contents of Ca^2+^ (**a**), water-soluble pectin (**b**), ionic-soluble pectin (**c**), covalent-soluble pectin (**d**), cellulose (**e**), and hemicellulose (**f**) in litchi pulp. Asterisks indicate significant differences between CaCl_2_-treated and control fruits (* *p* < 0.05, ** *p* < 0.01, *** *p* < 0.001). The data are presented as the mean ± SEM of three replicates.

The Ca^2+^ content in the CaCl_2_-treated pulp increased sharply on the first day, followed by a decline from day 1 to day 5. The Ca^2+^ content in the control pulp rose continuously during storage. Notably, the Ca^2+^ content in the CaCl_2_-treated pulp was substantially higher than that in the control pulp on days 1 and 3, whereas it was significantly lower than that in the control pulp on day 5 (Figure 4a).

The contents of WSP, ISP, CSP, and hemicellulose in both CaCl_2_-treated and control pulps showed a downtrend with increasing storage time, and the values in the CaCl_2_-treated pulp were consistently higher than those in the control pulp. The WSP, ISP, and CSP contents in the CaCl_2_-treated pulp declined sharply before day 1, while the contents in the control pulp declined sharply before day 3. Furthermore, the WSP, ISP, and CSP in the CaCl_2_-treated pulp were significantly higher than those in the control pulp on the days 3 and 5. A higher level of hemicellulose content was detected in the CaCl_2_-treated pulp, with prominent differences observed on day 5. CaCl_2_ treatment did not affect the cellulose content in the pulp (Figure 4b–f).

**Figure 5:**
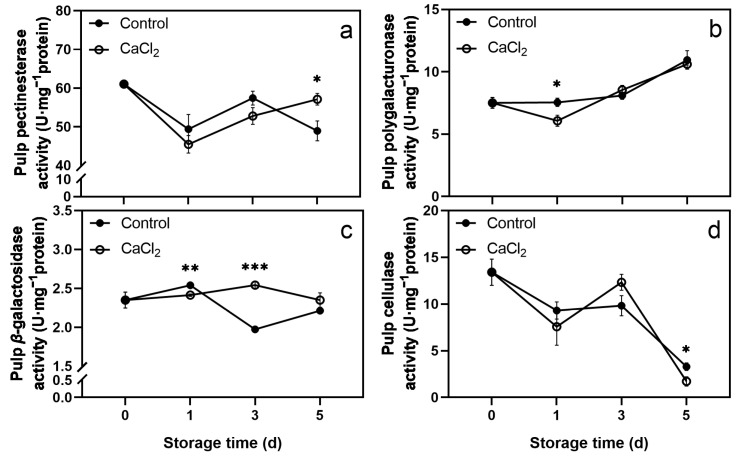
Effects of CaCl_2_ treatment on the activities of pectinesterase (**a**), polygalacturonase (**b**), *β*-galactosidase (**c**), and cellulase (**d**) in litchi pulp. Asterisks indicate significant differences between CaCl_2_-treated and control fruits (* *p* < 0.05, ** *p* < 0.01, *** *p* < 0.001). The data are presented as the mean ± SEM of three replicates.

The PE activity in both CaCl_2_-treated and control pulps displayed a downtrend during storage. The activity decreased on day 1 and increased during days 1–3. The difference was that the PE activity in the CaCl_2_-treated pulp continued to rise from day 3 to day 5, whereas the activity in the control pulp decreased. Therefore, the PE activity in the CaCl_2_-treated pulp was significantly higher than that in the control pulp on day 5 (Figure 5a). The PG activity in the CaCl_2_-treated pulp decreased slightly at on day 1 and then increased. The activity in the control fruit increased gradually during storage. Compared to the control pulp, a lower level of PG activity was observed in the CaCl_2_-treated pulp on day 1 (Figure 5b). The *β*-Gal activity in the control pulp increased slightly on the first day, followed by a rapid reduction on days 1–3. Compared to the control pulp, the CaCl_2_-treated pulp showed a lower *β*-Gal activity on day 1, and a higher *β*-Gal activity on day 3 (Figure 5c). The Cx activity reduced quickly in both the CaCl_2_-treated and control pulps. The activity in the CaCl_2_-treated pulp was significantly lower than that in the control pulp on day 5 (Figure 5d).

## 4. Discussion

### 4.1. CaCl_2_ Treatment Strengthened the Cell Wall Structure of Litchi Fruit

The methods involved in postharvest CaCl_2_ treatment include immersion treatment, vacuum infiltration treatment, and spray treatment. Since the pericarp and pulp of litchi completely separated in the structure, we used the vacuum infiltration method to enhance the effect of CaCl_2_ treatment. Figure S1 shows that the contents of titratable acid and ascorbic acid, respiration rate, and weight loss were not significantly different between CaCl_2_-treated and control fruits. Although CaCl_2_ treatment significantly reduced the total soluble solids content of litchi fruit on storage day 1, it had no significant impact on days 3 and 5. These results indicate that CaCl_2_ treatment did not cause apparent damage to the quality of litchi fruit. It is worth noting that CaCl_2_ treatment effectively delayed pericarp browning and decay (Figure 1), and the cell wall component contents and cell wall-degrading enzyme activities of litchi pericarp and pulp were changed (Figure 2, Figure 3, Figure 4 and Figure 5).

Ca^2+^ can cross-link unesterified homogalacturonan molecules via ionic calcium bridges to form a calcium–pectate gel, thereby increasing cell wall stiffness. Additionally, calcium–pectate cross-links in the middle lamella interlink most cells in ripe fruit [39]. Cell wall components include pectin, cellulose, and hemicellulose. WSP, ISP, and CSP are different forms of pectin. During the ripening and senescence process, ISP and CSP are converted to WSP, and the contents of cellulose and hemicellulose are decreased [36,40]. The cell wall components and structure are gradually degraded under the action of cell wall-degrading enzymes such as PE, PG, *β*-Gal, and Cx. Among them, PE, PG, and *β*-Gal act on pectin, and Cx acts on cellulose [36]. Many studies have confirmed the effect of postharvest CaCl_2_ treatment on the contents of cell wall components and the activities of cell wall-degrading enzymes in fruit. Yu et al. reported that the immersion of grape clusters in a 5 g L^−1^ CaCl_2_·2H_2_O solution containing 0.01% Tween 20 for 2 min inhibited WSP production, and delayed the degradation of protopectin (non-water-soluble pectin, including ISP and CSP) [22]. Liu et al. found that apricots treated with a 1% CaCl_2_ solution for 2 min reduced the degradation of WSP, CSP, and hemicellulose [27]. Chen et al. showed that strawberries treated with a 1% CaCl_2_ solution for 15 min slowed the breakdown of ISP and CSP [41]. Mohebbi et al. declared that dipping apples in a 4% CaCl_2_ solution for 5 min increased the contents of ISP and CSP, and decreased the activities of β-Gal, α-L-arabinofuranosidase, pectate lyase (PL) and PG [23]. Lv et al. reported that a 0.2 mol L^−1^ CaCl_2_ solution treatment maintained higher levels of protopectin and cellulose, lower levels of WSP, and decreased the activities of PG, pectin methylesterase (PME), Cx and *β*-Gal of red raspberry [24]. Gao et al. stated that papaya immersed in a 2.5% CaCl_2_ solution for 15 min repressed the activities of Cx, *β*-glucosidase, PG, and PME, and the expression of cell wall softening-related genes [25]. Zhang et al. explained that full exposure to a 0.18 mol L^−1^ CaCl_2_ solution for 25 min decreased the activities and gene expression of PG, PME and PL of New Queen melon [26]. These studies have illustrated that CaCl_2_ treatment maintains or strengthens the cell wall structure by inhibiting the production of WSP, the degradation of ISP, CSP, cellulose, and hemicellulose, as well as the activities of cell wall-degrading enzymes, thereby preventing fruit cracking and delaying fruit softening.

In our study, vacuum infiltration enhanced the effect of CaCl_2_ treatment, resulting in changes in the contents of cell wall components and in the activities of cell wall-degrading enzymes in both the pericarp and the pulp of litchi. Similarly to previous studies, in the pericarp, CaCl_2_ treatment significantly increased the contents of Ca^2+^ and cellulose while reducing the content of WSP and the activities of PG, *β*-Gal, and Cx. However, in the pulp, CaCl_2_ treatment significantly increased the contents of Ca^2+^, WSP, ISP, CSP, and hemicellulose, and the activities of PE and *β*-Gal, in addition to reducing the activities of PG and Cx. Thus, this study illustrates that the changes between the pericarp and pulp were different, and the CaCl_2_ treatment had a more pronounced effect on the pericarp. We believe this was related to the limitation of the treatment effect by the fruit structure, and the difference in the content of each cell wall component between the pericarp and the pulp. The content of each cell wall component in the pericarp was significantly higher than that in the pulp. Overall, CaCl_2_ treatment strengthened the cell wall structure of litchi fruit, greatly strengthened the firmness of the pericarp, and delayed the softening of the pulp to a certain extent.

### 4.2. CaCl_2_ Treatment Delayed the Pericarp Browning of Litchi

Enzymatic browning is one of the most frequent causes of litchi pericarp browning. Under normal circumstances, oxidative enzymes and their substrates exist in distinct subcellular compartments. The compartmentation limits enzymatic browning in the healthy pericarp [42]. We found that CaCl_2_ treatment maintained or strengthened the structure and function of the cell wall (Figure 2), and the firmness of the pericarp was strengthened. Therefore, the integrity of subcellular compartments remained and the litchi pericarp enzymatic browning was delayed. In addition, enzymatic browning was associated with oxidation resistance. CaCl_2_ treatment can decrease reactive oxygen species (ROS) content, and increase antioxidant enzyme activities and non-enzymatic antioxidant contents. For example, 80 mM CaCl_2_ significantly increased the activities of antioxidant enzymes peroxidase (POD), superoxide dismutase (SOD), and catalase (CAT) in the ‘Kyoho’ grapevine [15]. With the ‘Nanguo’ pear, the activity and gene expression of polyphenol oxidase (PPO) were inhibited, and the activity and gene expression of SOD were promoted under 2% CaCl_2_ treatment [18]. CaCl_2_ treatments maintained higher levels of phenolics, flavonoids, anthocyanins, ascorbic acid, and total antioxidant capacity of cornelian cherry [43]. A total of 10 g L^−1^ CaCl_2_ reduced the levels of superoxide anion and hydrogen peroxide, preserved the activities of SOD, CAT and POD, as well as the levels of ascorbic acid and glutathione of jujube [17]. However, the effect of CaCl_2_ treatment on the oxidation resistance of litchi pericarp requires further research.

### 4.3. CaCl_2_ Treatment Delayed the Decay of Litchi Fruit

Softening of the fruit is conducive to disease infestation. CaCl_2_ treatment effectively delays fruit ripening, senescence, and softening, which is closely related to the role of calcium in maintaining the cell wall [11]. CaCl_2_ treatment inhibited the softening of New Queen melon and papaya, slowed down the growth of microorganisms, and reduced the fruit decay rate [26,44]. CaCl_2_ treatment can induce disease resistance. In Hami melon, it resisted penicillium infection by causing calcium-dependent protein kinase (CDPK) activity and upregulating HmCDPK2 gene expression [33]. Moreover, CaCl_2_ can directly inhibit the growth of pathogenic microorganisms [45]. In our study, CaCl_2_ treatment significantly inhibited cell wall degradation in litchi pericarp and pulp in order to resist disease infestation.

## 5. Conclusions

CaCl_2_ treatment significantly strengthened the cell wall structure and delayed the cell wall degradation of litchi fruit. Therefore, pericarp browning, pulp softening, and disease incidence were inhibited, effectively maintaining the quality of litchi fruit. Since the effect of vacuum infiltration on the pulp is limited, further research on CaCl_2_ treatment without vacuum infiltration can be considered for production applications.

## Data Availability

Data is contained within the article.

## Supplementary Materials

The following supporting information can be downloaded at: https://www.mdpi.com/article/10.3390/foods12132478/s1, Figure S1: Effects of CaCl_2_ treatment on total soluble solids content (a), titratable acid content (b), ascorbic acid content (c), respiration rate (d), and weight loss (e).
