# Supplementary material for: Postharvest Calcium Chloride Treatment Strengthens Cell Wall Structure to Maintain Litchi Fruit Quality
Source: Foods. 2023 Jun 25;12(13):2478. doi: 10.3390/foods12132478 (PMC10340422; doi:10.3390/foods12132478)
Supplement: Supplementary file 1 [file foods-12-02478-s001.zip › foods-2443085-supplementary.pdf]

Evaluation Method for Pericarp Browning Index and Decay

Pericarp browning was evaluated based on the browned area on each fruit pericarp, using the following grades: Grade 0, no browning or a few sporadic brown spots on the pericarp; Grade 1, distinct brown spots on the pericarp; Grade 2, the browning area covers less than 1/3 of the pericarp, and each brown patch is less than 0.5 cm^2; Grade 3, the browning area covers 1/3–1/2 of the pericarp, and each brown patch is 0.5 cm^2–1 cm^2; Grade 4, the browning area covers 1/2–3/4 of the pericarp, and each brown patch is more than 1 cm^2; and Grade 5, the browning area covers more than 3/4 of the pericarp. The pericarp browning index of each replication was calculated as follows:

$$Pericarp\ browning\ index = \frac{\sum\ (Browning\ grade\ \times\ Number\ of\ fruit\ in\ each\ grade)}{Total\ number\ of\ fruit}$$

The decay of each replication was calculated as follows:

$$Decay = \frac{Fruit\ with\ mycelia\ and\ rot}{Total\ number\ of\ fruit}$$

Methods for the Data in Figure S1

1. Determination of Total Soluble Solid (TSS) Content and Titratable Acid (TA) Content

The pulp was juiced and determined the contents of TSS and TA using a refractometer (PAL-BX|ACID5, ATAGO, Tokyo, Japan), according to the manufacturer's instructions.

2. Determination of Ascorbic Acid Content

The pulp was juiced and diluted 100 times with 2% oxalic acid solution (pH6). The absorbance was measured at 267 nm. The ascorbic acid content was calculated according to equation:

$$\text{ascorbic acid content } (\mu\text{L} \cdot \text{mL}^{-1}) = \frac{\text{m}}{50} \times 100$$

m: the content of ascorbic acid obtained from the standard curve is equivalent to the quality of ascorbic acid contained in a 50 mL volumetric flask.

3. Determination of Respiration Rate

Twenty fruits were randomly selected and placed in a hermetically sealed box for 2 h at 25 °C ± 2 °C. The CO_2 concentration in the box was determined using a gas chromatograph (GC7900, Techcomp, China). The conditions were set as follows: carrier gas, He; current: 30 mA; column temperature, 50 °C; injection port temperature: 120 °C; thermal conductivity detector (TCD) temperature, 150°C. The respiration rate for the various durations was measured in triplicate samples and calculated according to equation:

$$\text{Respiration rate } (\text{mg} \cdot \text{kg}^{-1} \cdot \text{h}^{-1}) = \frac{\text{A} \times (\text{V}_1 - \text{V}_2) \times \text{M} \times 273}{\text{H} \times \text{W} \times 22.4 \times (273 + \text{T})}$$

A: CO_2 concentration (%); V_1: volume (mL) of the hermetically sealed box; V_2: the total volume (mL) of the fruits; M: the molar mass of CO_2 (44 mg $mmol^{-1}$) ; H: the time in the sealed environment (h); W: the total fruit weight (kg); T: the storage temperature (°C).

4. Determination of Weight Loss

Sixty fruits were randomly selected for each treatment to evaluate the weight loss. The weight loss calculated according to equation:

$$\text{weight loss (\%)} = \frac{\text{weight}_{(i)} - \text{weight}_{(t)}}{\text{weight}_{(i)}} \times 100\%$$

$\text{weight}_{(i)}$: the initial weight of fruits; $\text{weight}_{(t)}$: the weight of fruits on time t.

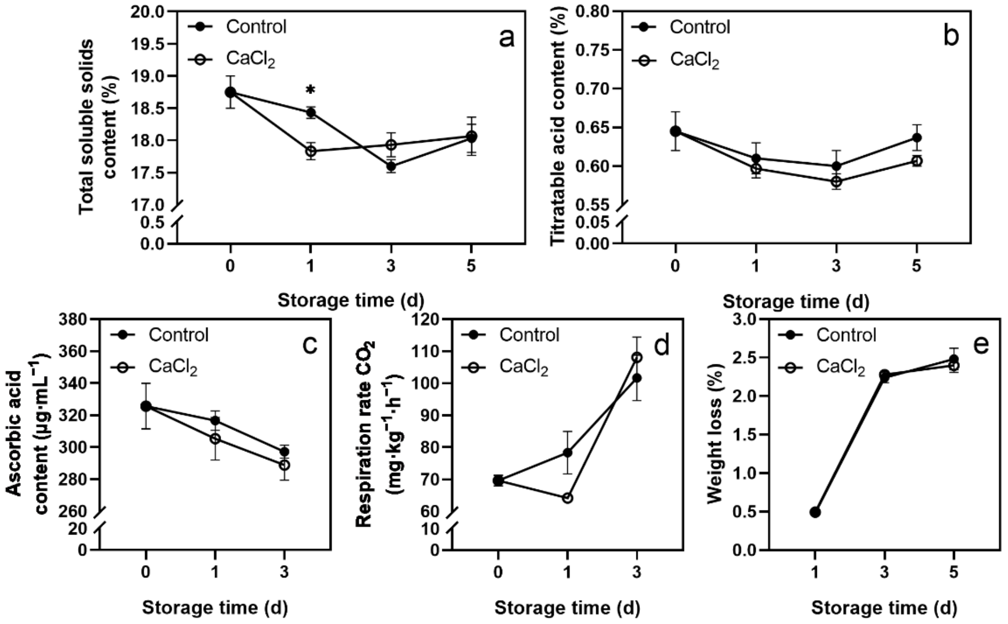

Figure S1. Effects of $CaCl_2$ treatment on total soluble solids content (**a**), titratable acid content (**b**), ascorbic acid content (**c**), respiration rate (**d**), and weight loss (**e**). Asterisks indicate significant differences between $CaCl_2$-treated and control fruits (*$p < 0.05$). The data are presented as the mean ± SEM (standard error of the mean) of three replicates.
